# **In Patients with** Minor Beta-Thalassemia, Cognitive Performance Is Related to Length of Education, But Not to Minor Beta-Thalassemia or Hemoglobin Levels

**Published:** 2019-01

**Authors:** Mohammad Ahmadpanah, Yasaman Asadi, Mohammad Haghighi, Hamidreza Ghasemibasir, Elham Khanlarzadeh, Serge Brand

**Affiliations:** 1 Research Center for Behavioral Disorders and Substance Abuse, Hamadan University of Medial Sciences, Hamadan, Iran.; 2 Department of Pathology, School of Medicine, Hamadan University of Medical Sciences, Hamadan, Iran.; 3 Department of Community Medicine, School of Medicine, Hamadan University of Medical Sciences, Hamadan, Iran.; 4 University of Basel, Psychiatric Clinics, Center for Affective, Stress and Sleep Disorders, Basel, Switzerland.; 5 University of Basel, Department of Sport, Exercise and Health, Division of Sport Sciences and Psychosocial Health, Basel, Switzerland.; 6 Kermanshah University of Medical Sciences, Sleep Disorders Research Center and Substance Abuse Prevention Research Center, Kermanshah, Iran.

**Keywords:** *Beta-Thalassemia Minor*, *Cognitive Performance*, *Long-Term Memory*, *Length of Education*, *Working Memory*

## Abstract

**Objective:** Thalassemia is one of the most frequent monogenic disorders, leading to impairment in the maturation and survival of red blood cells. The question examined here is whether, and if so, to what extent, people with beta-thalassemia might also be impaired in their cognitive functioning. Previous results in adults with beta-thalassemia showed cognitive impairment when compared to healthy controls. However, length of education was never taken into consideration as a possible confounder. Accordingly, the aim of the present study was to assess people with minor beta-thalassemia and compare them to healthy controls, while controlling for length of education.

**Method** **:** A total of 25 adults (mean age: 29.36 years; 56% females) with beta-thalassemia and 25 healthy controls (mean age: 27.84 years; 72% females) took part in this cross-sectional study. They underwent cognitive testing (executive functions, attention, working memory), and their haemoglobin levels were assessed.

**Results: **Cognitive performance did not significantly differ between patients with minor beta-thalassemia and healthy controls. Irrespective of group, higher cognitive performance was strongly associated with time spent in education. No gender differences were observed.

**Conclusion: **Compared to healthy controls, cognitive performance was not impaired among patients with minor beta-thalassemia when length of education was introduced as a further co-variate. In both patients with minor beta-thalassemia and healthy controls, higher cognitive performance was associated with time spent for education. Health professionals should inform patients with minor beta-thalassemia that cognitive performance is related to the length of education and not to the health status of minor beta-thalassemia per se.

Thalassemia is a monogenic disorder, which passes from parents to their offspring by autosomal recessive inheritance (1, 2). Further, thalassemia is a heterogeneous group of abnormalities in haemoglobin biosynthesis, which is particularly prevalent in Mediterranian area (Italy, Cyprus, Greece, Sicily), the Middle East (Iran, Turkey, Syria), and Southeast Asia, an area thus extending from southwest Europe to the Far East. 

The main defect is not in the molecular structure of haemoglobin; rather, the abnormality is in the quantitative synthesis of each alpha and beta chain, resulting in an unbalanced synthesis of globin chains and in the precipitation of additional chains, which ultimately causes impairment in the maturation and survival of red blood cells and of the red blood cell lysis (1-3(.

Beta-thalassemia is also found in vast areas of Central Africa 4. 

Thalassemia is one of the most common genetic disorders in the world, with approximately 200 million sufferers. About 15% of African-Americans are thalassemia carriers. The trait of Alpha thalassemia (minor) is prevalent in 3% of the Americans and in 1%-15% of the Mediterranean population (3). Each year, more than 4 million children in the world are born with genetic disorders, and thalassemia is one of the most prevalent type of these disorders (5, 6). Transfusion and iron chelation are the first-line treatments for this disorder, while efforts are also now being made to treat thalassemia via gene therapy (7).

Given the high prevalence of thalassemia and the challenges posed by its treatment, the question arises whether, and if so to what extent, minor beta-thalassemia might be associated with poorer cognitive functioning. Previous works have compared children and adults with beta-thalassemia to gender- and age-matched controls. For example, Shehata et al. (8) compared cognitive functions and thalassemia biomarkers, such as ferritin, serum transferrin receptor, and nitric oxide levels, in 40 children with thalassemia and 40 healthy controls. As expected, significantly higher levels of ferritin and serum transferrin receptors with decreased nitric oxide were detected among children with beta-thalassemia. Cognitive functions did not differ between children with and without thalassemia; however, there were significant correlations between serum transferrin receptors and nitric oxide levels on the one hand and cognitive functioning on the other. In contrast, Duman et al. (9) found a significant reduction in the executive performance and verbal IQ of children with major beta-thalassemia when compared to healthy controls. However, Karimi et al. (10) compared the IQ (Raven’s Test) of children with major beta-thalassemia and of healthy children, but found no difference. 

As regards to adults, Nevruz et al. (11) showed that the cognitive functioning of 32 patients with beta-thalassemia was poorer than that of controls. Zangiabadi et al. (12) compared the cognitive functioning of 30 patients with beta-thalassemia with that of gender- and age-matched controls and showed that the former had lower performances on the vocabulary and arithmetic subtests of the Wechsler Adult Intelligence Scale (WAIS). Monastero et al. (13) investigated cognitive performance decline and focused on abstract reasoning, attention, executive functions, language, constructional/visuospatial skills, and memory. Their sample consisted of 46 patients with (major) beta-thalassemia and 46 healthy controls. The patient group had lower scores for cognitive performance than the healthy controls. Poorer cognitive performance was also associated with a shorter time interval between the onset of blood transfusions, while the onset of chelating treatment correlated with performances on tests assessing abstract reasoning, attention, constructional/visuospatial skills, and memory. However, cognitive performance and haemoglobin levels were unrelated (13).

To summarize, evidence on the association between beta-thalassemia and cognitive performance is not consistent. While no such association has been found among children, research on adults has indicated an association between beta-thalassemia status and cognitive performance, but no association has been observed between haemoglobin levels and cognitive performance. 

Strangely, to our knowledge, no study has yet considered gender and educational level as factors. Given the extensive body of research on sex differences in physiological and psychological characteristics, such differences may also be observable as regards to beta-thalassemia and cognitive performance. Research shows that the extent and level of education impacts on later cognitive performance (14, 15). Thus, it is likely that current cognitive performance reflects previously accumulated exposure to education. 

The following hypotheses and one research question were formulated. First, we anticipated that haemoglobin levels would be higher in people with minor beta-thalassemia than in healthy controls. Second, following previous results (11-13), we expected poorer cognitive performance in adults with minor beta-thalassemia than in healthy controls. Third, following Shehata et al., 8 we expected that poorer cognitive performance and higher beta-thalassemia biomarkers would be associated. Fourth, following others (14, 15), we expected that current cognitive performance would be associated with years of education completed. Next, we treated as exploratory whether and to what extent gender differences might be observed.

We believe that this study has the potential to shed light on the complex association between minor beta-thalassemia and cognitive performance, while considering beta-thalassemia-related biomarkers along with possible confounders such as gender and education.

## Materials and Methods

***Procedure***

Eligible patients with minor beta-thalassemia and healthy controls were approached to participate in the study. All participants were informed about the aims of the study and the anonymous data handling. Then, they signed a written informed consent. Participants completed questionnaires on sociodemographic and health-related information. Next, they underwent cognitive testing (cognitive flexibility; performance attention; information processing; working memory). Blood samples were taken between 8 am and 9 am. The study was approved by the Review Board of Hamadan University of Medical Sciences (HUMS; Hamadan, Iran) and performed in accordance with the rules of the Declaration of Helsinki and its later amendments.

***Samples***

Adults with minor beta-thalassemia:

Individuals with known minor beta-thalassemia undergoing risk-analysis for marriage were asked to participate in the study. A total of 41 people was approached and 25 (61%) agreed to participate. Inclusion criteria were as follow: (a) age 18-40 years; (b) willing and able to follow the study protocol; (c) written informed consent; (d) known and medically confirmed minor beta-thalassemia. Exclusion criteria were as follow: (a) known and confirmed mental impairments, such as psychiatric disorders (eg, major depressive disorders, bipolar disorders, substance use disorders, neurodegenerative disorders, autism spectrum disorders, schizophrenia), or mental retardation; (b) acute suicidality; (c). Illiteracy. 

***Healthy Controls***

Healthy controls were recruited from hospital staff members and their adult relatives. Inclusion criteria were as follow: (a) age 18- 40 years; (b) willing and able to follow the study protocol; (c) written informed consent. Exclusion criteria were as follow: (a) known and medically confirmed minor beta-thalassemia; (b) Current psychiatric issues (eg, major depressive disorders, bipolar disorders, substance use disorders, neurodegenerative disorders, autism spectrum disorders, schizophrenia, and acute suicidality); (c) Current somatic issues. 

***Tools***

***Sociodemographic Information***

Participants completed a questionnaire providing details of age, gender, and education. 

***Assessing Executive Performance with the Wisconsin Card Sorting Test***

As described elsewhere(        16 ), the Wisconsin card sorting test (WCST) is a neuropsychological test to assess cognitive flexibility or, more specifically, the ability to display flexibility in the face of changing schedules of reinforcement (17, 18). First, a number of stimulus cards are presented to the participant. Next, the experimenter tells the respondent to match the cards and tells her/him whether a particular match is right or wrong. The respondent should gain insight into the hidden pattern (rule) and match the cards accordingly. Following an algorithm unknown to the respondent, the hidden pattern (rule) is changed, and he or she needs to discover the new hidden pattern (rule). From the perspective of cognitive psychology, three cognitive tasks have to be solved: first, gaining insight into the hidden pattern; second, building a cognitive representation of the hidden pattern; and third, shifting from this cognitive representation to a new insight into how to solve the now modified problem (eg, achieving freedom from functional fixation). The test takes approximately 12–20 minutes to complete and generates a number of psychometric measures, including numbers, percentages, and percentiles of categories achieved, trials, errors, and perseverative errors. 

***Assessing performance attention***

The digit span subtest of the Paced Auditory Serial Addition Test (PASAT) were used to assess performance attention. 

As outlined elsewhere (   19 ), the PASAT (20, 21) is a test battery to assess auditory information processing speed and flexibility. Single digits are presented either every 3 seconds (3-second PASAT) or every 2 seconds (2-second PASAT), and the respondent must add each new digit to the one immediately prior to it. The test result is the number of correct sums given out of the 60 possible correct answers. 

***Assessing Working Memory***

The Digit Span subtest of the Wechsler (Wechsler Adult Intelligence Scale) was used to test working memory. Briefly, in the Digit Span subtest one has to repeat a series of numbers in the same order given, backwards, or from lowest to highest.

***Haemoglobin Testing***

Electrophoresis was performed using automatic Interlab G26 by gel electrophoresis, along with a quantitative measurement of HbA2 by column chromatography using a bio-system kit. By combining these 2 methods, the exact amounts of HbA, HbA2, and HbF were determined to detect minor beta-thalassemia.

***Data Analysis***

A series of ANOVAs were computed with the factors of gender (male vs. female) and group (minor beta-thalassemia vs. healthy controls) and with cognitive tests, haemoglobin levels, and education (in years) as dependent variables. Next, a series of Pearson’s correlations were performed between cognitive tests, haemoglobin levels, and education, both for the entire group and separately for patients with minor beta-thalassemia and healthy controls. The nominal level of significance was set at alpha 0.05. Data analysis was performed with SPSS® 25.0 (IBM Corporation, Armonk NY, USA) for Apple® Mac®.

## Results

All descriptive and inferential statistical indices are reported in Tables 1 to 3. Accordingly, these statistics are not repeated in the text. 

***Sociodemographic Information***

Patients and healthy controls did not differ as regards to gender distribution, age, or years of education.

***Haemoglobin Levels***

Haemoglobin levels were significantly higher in the minor beta-thalassemia group than in the healthy controls, irrespective of gender (Table 1).

**Table 1:** Descriptive and Inferential Statistical Overview of Psychosocial and Blood-Related Information for People with and without Minor Beta-Thalassemia and for Gender

	**Groups**					**Statistics**
	**With Minor Beta-** **Thalassemia**			**Healthy Controls**		
	**Females**	**Males**		**Females**	**Males**	
N	14	11		18	7	X^2^(N= 50, df = 1) = 1.39, p = 0.24
	M (SD)	M (SD)		M (SD)	M (SD)	
Age (years)	29.57 (4.21)	29.09 (5.32)		27.67 (4.72)	28.29 (5.25)	Group: F(1, 45) = 0.88, p = 0.36 Gender: F(1, 45)= 0.01, p = 0.96 Group x Gender: F(1, 45)= 0.14, p = 0.71
Years of education	15.21 (2.60)	14.70 (2.50)		14.83 (3.24)	13.57 (1.99)	Group: F(1, 45) = 0.88, p = 0.38 Gender: F(1, 45)= 1.11, p = 0.29 Group x Gender: F(1, 45)= 0.99, p = 0.66
HbA1c	5.71 (0.55)	6.02 (0.41)		3.98 (1.26)	4.56 (1.60)	Group: F(1, 45) = 34.59, p = 0.001 Gender: F(1, 45)= 2.56, p = 0.19 Group x Gender: F(1, 45)= 1.18, p = 0.28

**Table 2:** Descriptive Overview of Cognitive Performance, Separately for Those with and without Minor Beta-Thalassemia and for Males and Females

			**Groups**	
	**Minor Beta-Thalassemia**		**Healthy Controls**	
	**Females**	**Males**	**Females**	**Males**
N	14	11	18	7
	M (SD)	M (SD)	M (SD)	M (SD)
WCST				
Classification	2.36 (2.02)	1.36 (1.12)	2.39 (2.40)	2.00 (1.63)
Perseveration	9.93 (7.33)	9.09 (9.63)	10.28 (7.03)	10.86 (6.04)
Conceptual	2.07 (2.70)	2.00 (2.72)	2.33 (3.01)	1.86 (2.85)
PASAT				
3 seconds	35.93 (15.61)	39.45 (11.26)	36.89 (12.79)	39.57 (11.72)
2 seconds	25.5 (11.27)	31.36 (9.04)	28.00 (11.86)	25.86 (12.27)
WAIS				
Coding	55.36 (14.87)	44.55 (15.74)	54.22 (15.31)	44.43 (6.32)
Forward	5.14 (2.28)	6.09 (2.91)	4.39 (2.00)	4.28 (1.80)
Backward	6.29 (1.90)	6.18 (2.40)	7.70 (2.78)	7.57 (1.99)

Notes: WCST = Wisconsin Card Sort Test; PASAT = Paced Auditory Serial Addition Test; WAIS = Wechsler Adult Intelligence

**Table 3:** Inferential Statistical Indices of Cognitive Performance with the Factors Group (with and without Minor Beta-Thalassemia), Gender, and Group by Gender Interaction

		**Factors**	
	**Group**	**Sex**	**Group x Sex Interaction**
	**F ** **partial eta** ^2^	**F ** **Partial eta** ^2^	**F ** **Partial eta** ^2^
WCST			
Classification	0.32 0.07	1.35 0.03	0.26 0.01
Perseveration	0.21 0.01	0.00 0.00	0.10 0.00
Conceptual	0.01 0.00	0.01 0.00	0.06 0.00
PASAT			
3 seconds	0.02 0.00	0.61 0.00	0.01 0.00
2 seconds	0.20 0.00	0.31 0.00	1.42 0.02
WAIS			
Coding	0.02 0.00	3.56 0.11	0.01 0.00
Forward	3.48 0.07	0.38 0.01	0.59 0.01
Backward	3.92 0.08	0.03 0.00	0.00 0.00

**Table 4:** Correlations of Age, Educational Level, and Haemoglobin Levels with Cognitive Performances

	**Age**	**Educational Level**	**Haemoglobin Level**
WCST			
Classification	-0.24^(*)^	0.43**	-0.06
Perseveration	0.27	-0.40**	-0.05
Conceptual	-0.24^(*)^	0.29*	-0.06
PASAT			
3 seconds	-0.18	0.54***	0.00
2 seconds	-0.14	0.52***	0.08
WAIS			
Coding	-0.39*	0.66***	0.06
Forward	-0.03	0.38**	0.08
Backward	-0.25^(*)^	0.20*	0.12

Notes: WCST = Wisconsin Card Sort Test; PASAT = Paced Auditory Serial Addition Test; WAIS = Wechsler Adult Intelligence; < 0.10;

(*) = p

* = p < 0.05,

** = p < 0.01;

*** = p < 0.001.

No significant differences were found in cognitive performances between the groups, genders, or as a function of the group by gender interaction (Tables 2 and 3).

***Correlations among Cognitive Performance, Age, Haemoglobin, and Educational level***

Table 4 demonstrates the intercorrelations among the dimensions of cognitive performance, age, haemoglobin, and educational level. 

Cognitive performance was unrelated to age or haemoglobin levels, though it was significantly related to years of education, both across the sample as a whole (Table 4) and separately for those with beta-thalassemia and healthy controls (data not shown). 

## Discussion

The key finding of this study was that patients with minor beta-thalassemia did not display impaired cognitive performance compared to healthy controls. Furthermore, cognitive performance was unrelated to either hemoglobin levels or gender. Years of education were strongly associated with level of cognitive performance. 

Four hypotheses and 1research question were formulated.

Our first hypothesis was that individuals with minor beta-thalassemia would have higher hemoglobin levels than healthy controls, and this was confirmed. While the result is unsurprising, it helped us ascertain that people with minor beta-thalassemia could be clearly distinguished from healthy controls at a biological level. 

Our second hypothesis was that individuals with minor beta-thalassemia would perform more poorly on cognitive tasks than healthy controls. This hypothesis was not supported. Thus, the present results are at odds with previously published findings for adults (11-13). However, our findings are consistent with evidence from studies of children (8, 10), which revealed no deficits in cognitive functioning associated with beta-thalassemia. We do not have the data that might provide a deeper understanding of the lack of difference we found, although in previous studies (11-13) a straightforward psychophysiological rationale was missing. Nevruz et al. (11) assessed cognitive performance with an auditory test, while they were not interested in the test performance. However, in the P300 potentials, Zangiabadi et al. (12) found lower performances in patients with minor beta thalassemia in subtests of arithmetic and vocabulary and picture completion, while on the non-verbal scale, they found no significant difference between the two groups. However, Zangiabadi et al. (12) and Monastero et al. (22) did not control for length of education.

Our third hypothesis was that a poorer cognitive performance would be associated with higher beta-thalassemia biomarkers, but again this hypothesis was not supported, and our findings contrast with evidence from previous research on children (8). On the other hand, the present findings confirm what Monastero et al. (13) observed among adults. Overall, the present results indicate that the associations between minor beta-thalassemia and its underlying blood-related and physiological processes and cognitive performance are not straightforward. 

Our fourth hypothesis was that current cognitive performance would be associated with length of time spent in education, and this hypothesis was strongly supported. We followed previous research (14, 15) showing that length of education has an impact on higher cognitive performance in later years of adulthood. While we have no direct evidence for this kind of causal relationship in the present study, it does seem possible that a more extended academic education could lead to greater cognitive flexibility, better knowledge, and superior performance later on. 

In response to the exploratory question, we found that gender was not a confounder, as cognitive performance, haemoglobin levels, duration of academic education did not systematically vary as a function of gender.

## Limitations

Despite the potential interest of the findings, there are some limitations that should be acknowledged. First, the sample size was rather small, although the means and standard deviations shown in Table 2 indicate that even with larger sample sizes significant differences would not have been found. Second, we focused on executive functions, performance attention, and working memory, while other domains of cognitive performance, such as problem solving, decision-making, language, and complex perception, might have yielded a different pattern of results. Third, it is possible that further unassessed latent variables might have biased 2 or more variables in the same or opposite direction. Accordingly, future studies might assess other cognitive, but also emotional domains, such as anxiety, coping, depression, and self-efficacy, as further possible targets.

## Conclusion

Among a sample of adults with minor beta-thalassemia and compared to healthy controls, level of cognitive performance was related to length of education, but not to haemoglobin levels or gender.
